# Submerged Fermentation of the Edible Mushroom *Pleurotus ostreatus* in a Batch Stirred Tank Bioreactor as a Promising Alternative for the Effective Production of Bioactive Metabolites

**DOI:** 10.3390/molecules17032714

**Published:** 2012-03-06

**Authors:** Lefki-Maria Papaspyridi, Nektarios Aligiannis, Evangelos Topakas, Paul Christakopoulos, Alexandros-Leandros Skaltsounis, Nikolas Fokialakis

**Affiliations:** 1 BIOtechMASS Unit, Biotechnology Laboratory, School of Chemical Engineering, National Technical University of Athens, 9 Iroon Polytechniou Street, Zografou Campus, GR-15700 Athens, Greece; Email: leriapap@mail.ntua.gr (L.-M.P.); vtopakas@central.ntua.gr (E.T.); hristako@chemeng.ntua.gr (P.C.); 2 Department of Pharmacognosy and Natural Products Chemistry, Faculty of Pharmacy, University of Athens, Panepistimioupolis, GR-15771 Athens, Greece; Email: aligiannis@pharm.uoa.gr (N.A.); skaltsounis@pharm.uoa.gr (A.-L.S.)

**Keywords:** *Pleurotus ostreatus*, submerged fermentation, stirred tank bioreactor, bioactive metabolites, fast centrifugal partition chromatography

## Abstract

The aim of this study was to investigate the potential of the submerged fermentation procedure in the production of bioactive metabolites of the common edible mushroom *Pleurotus ostreatus.* The biomass of the mushroom strain was produced by submerged fermentation in a batch stirred tank bioreactor and extracted by solvents of increasing polarity. The dichloromethane and methanol extract were fractioned by different techniques including Adsorption Chromatography and Fast Centrifugal Partition Chromatography (FCPC). The structures of pure compounds were elucidated with 1D/2D NMR-spectroscopic analyses, and chemical correlations combined with GC/MS and LC/MS experiments. Nineteen metabolites (e.g., fatty acids, phenolic metabolites, nucleotides and alkaloids) were isolated. Beyond the production of known metabolites, we report herein the production also of *trans*-3,4-dihydro-3,4,8-trihydroxynapthalen-1(2*H*)-one, indolo-3-carboxylic acid, 3-formylpyrrole and 4-hydroxybenzoic acid, that have pharmaceutical interest and are isolated for the first time from *Pleurotus* strains.This work indicates the great potential of the established bioprocess for the production of *P. ostreatus* mycelia with enhanced metabolic profile.

## 1. Introduction

Many cultures worldwide have long recognized that beyond their nutritional value certain mushrooms can have some health-promoting benefits as well. In China and Japan, in particular, many of these mushrooms have become important ingredients in Traditional Chinese Medicine. At least 270 species of mushrooms are considered to possess therapeutic properties [1], and nowadays the term “medicinal mushrooms” has gained worldwide recognition. Many edible mushrooms used in traditional folk medicine, including *Lentinus edodes* (shiitake mushroom), *Grifola frondosa* (maitake), *Hericium erinaceus*, *Flammulina velutipes*, *Tremella mesenterica* and *Pleurotus ostreatus* are considered a good source of bioactive compounds [2].

*P. ostreatus*, also known as the oyster mushroom, is a Basidiomycetes belonging to the family Pleurotaceae (Agaricales, Agaricomycetes). Interest in this species has increased considerably in the last decade because of its gastronomic value and its nutraceutical properties [3]. The medicinal beneficial effects of *P. ostreatus*, such as antioxidant, antitumor and cholesterol-lowering activities, have been investigated intensively [4].

Currently, commercial mushroom products are mostly derived from the fruitbodies of field-cultivated mushrooms, which is a time-consuming and labor-intensive process. Submerged cultivation of edible and medicinal mushrooms has received increasing attention around the world and is viewed as a promising alternative for efficient production of biomass and valuable metabolites. Specifically, it offers potential advantages of faster production for both mycelia biomass and metabolites, in a shorter time period within reduced space and lesser chances for contamination [5].

Considering the great interest for mushrooms as a source of bioactive metabolites for the development of drugs and nutraceuticals, the objective of this study was the chemical investigation of a *P. ostreatus* commercial strain that we have previously optimized in terms of biomass production in a pilot scale [6]. This study presents the isolation and identification of compounds derived from biomass by submerged fermentation of the studied strain in stirred tank bioreactor. The information obtained in this work is considered fundamental and useful for the further development of the studied higher fungi fermentation process on an industrial scale, for enhanced bioactive metabolites production.

## 2. Results and Discussion

**Figure 1:**
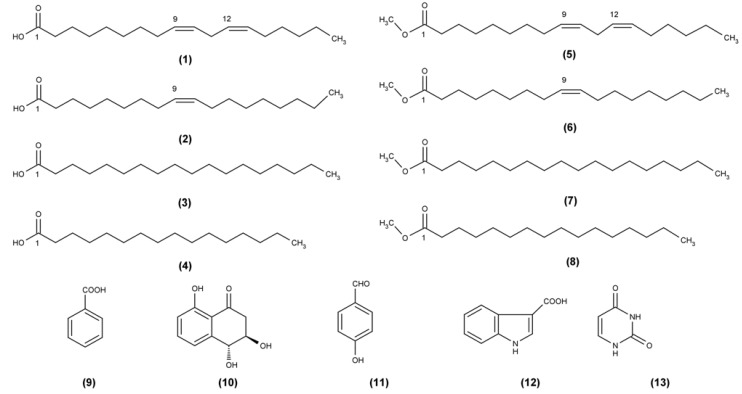
Structures of metabolites isolated from the dichloromethane (DCM) extract of biomass derived from *P. ostreatus* grown in submerged culture in a batch stirred tank bioreactor.

The biomass of *P. ostreatus* was produced by submerged fermentation in a batch stirred tank bioreactor and subsequently extracted with DCM and MeOH. The initial fractionation of the crude dichloromethane (DCM) extract of biomass was performed by preparative MPLC. The investigation of the resulting non-polar fractions by GC/MS analysis afforded the identification of four fatty acids and their derivatives. Specifically, the above investigation afforded linoleic acid (**1**), oleic acid (**2**), stearic acid (**3**), palmitic acid (**4**) and their corresponding methyl esters (**5**, **6**, **7** and **8**, respectively) (Figure 1). 

Further chromatographic seperation of the rest of the combined fractions obtained by the DCM extraction, using MPLC and Sephadex LH-20 column chromatography, led to the identification of benzoic acid (**9**) [7], *trans* 3,4-dihydro-3,4,8-trihydroxynapthalen-1(2H)-one (**10**) [8], 4-hydroxy-benzaldehyde (**11**) [9], indolo-3-carboxylic acid (**12**) [10] and uracil (**13**) [11] (Figure 1).

Based on existing literature data, all the isolates obtained from the fractionation and investigation of the DCM extract may be regarded as functional food ingredients or as constituents of great interest to the pharmaceutical industry, exhibiting numerous health benefits such as antiviral, antitumor and hypocholesterolemic activities [12,13,14,15,16,17,18]. Interestingly, the presence of linoleic (**1**), oleic (**2**), stearic (**3**) and palmitic acid (**4**) in the mycelium produced in a batch stirred bioreactor, which have been anyway found in naturally occurring fruitbodies of *P. ostreatus* [19], indicates that the established bioprocess does not prevent the production of these main fatty acids. Similarly, the submerged fermentation seems to not prevent the production of benzoic acid (**9**), a phenolic compound which has been extracted before from the fruitbody of *P. ostreatus* and that also exerts antibacterial activities [20]. 

**Figure 2:**
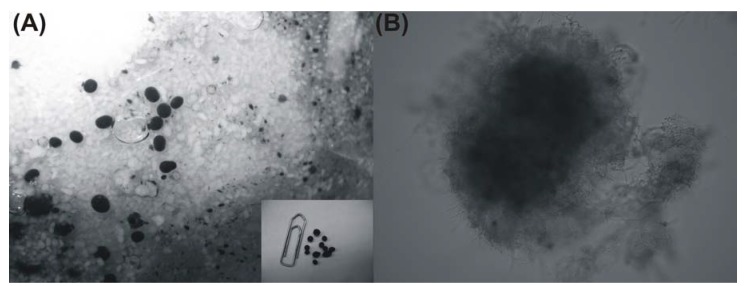
(**A**) Darkly pigmented pellets as they appear in the culture broth and (**B**) heavily melanized pellet formed by submerged fermentation of *P. ostreatus* in a batch stirred tank bioreactor as seen through an optical microscope (magnitude 40×).

On the other hand, polyhydroxylated α-tetralones, such as *trans-*3,4-dihydro-3,4,8-trihydroxy-napthalen-1(2*H*)-one (**10**), are known as metabolites implicated in the branched pathway of fungal DHN-melanin biosynthesis [21]. It should be cited that melanins are pigments of high molecular weight formed by oxidative polymerization of phenolic or indolic compounds and usually are dark brown or black. Fungal melanins are conferring certain advantages to fungi such as increasing their survival potential in some environments and enhancing their virulence [22]. *Pleurotus cystidiosus* has been reported to produce darkly pigmented arthroconidia forming a black pigment on mycelium or basidiomata [23]. The black pigment was confirmed as melanin and characterized [24]. Similar black pigments formed during the cultivation of *P. ostreatus* in this study (Figure 2A).

Specifically, fungal pellets were heavily melanized, while the optical microscopy of the material revealed a microfibrillar structure (Figure 2B). Interestingly, melanin production by *P. ostreatus* was induced when the growth rate declined, as the melanin formation commenced after the termination of the logarithmic growth phase (data not shown).

The presence of *trans*-3,4-dihydro-3,4,8-trihydroxynapthalen-1(2*H*)-one (**10**) in *P. ostreatus* is reported in this work for the first time. As for 4-hydroxybenzaldehyde (**11**), it has been previously detected in liquid cultures of Pleurotus species, including *P. ostreatus* [25], under different cultivation conditions.

Regarding indole-3-carboxylic acid (**12**), although it was formerly isolated from many macromycetes, with the most recent report being in the Ascomycetes *Gaeumannomyces amomi* [26], it has not been detected in *P. ostreatus* before. Interestingly, secondary products of fungi, which contain the indolic moiety commonly use as a biosynthetic precursor tryptophane [27], which was the most stimulatory amino acid when used as nitrogen source in *P. ostreatus* liquid culture [6]. Besides, this amino acid is present in the nitrogen source used for the biomass production in this study (corn steep liquor).

The extraction of phenolic compounds of the methanol (MeOH) extract was performed by adsorption-desorption processes using XAD4 type resin. The chemical structure of the resin material favored adsorption by weak interactions of molecules with moieties of high electron density, such as aromatic rings. In contrast, sugars or polar lipids couldn’t establish this kind of interaction and were eluted with water flow during the rinsing phase. The adsorbed phenolic compounds were recovered by elution with MeOH, giving an enriched extract. From this procedure it was indicated that almost 14% of the methanolic extract consists of phenolic compounds.

**Figure 3:**
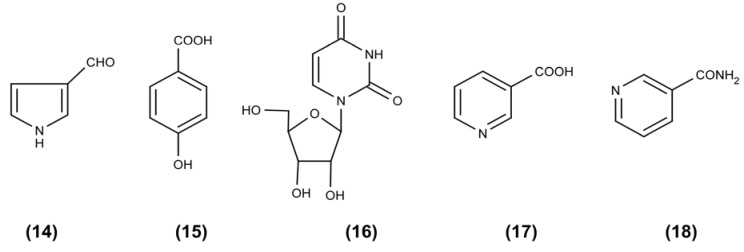
Structures of metabolites isolated from the phenolic fraction of the methanolic (MeOH) extract derived from *P. ostreatus* grown in submerged culture in a batch stirred tank bioreactor.

The investigation of the phenolic extract, based on the effective fractionation by preparative FCPC (separation), afforded 3-formyl-pyrrole (**14**) [28], 4-hydroxy-benzoic acid (**15**) [29], uridine (**16**) [30], nicotinic acid (**17**) and nicotinamide (**18**) (Figure 3).

In addition, all isolated compounds obtained by the fractionation and investigation of the phenolic fraction of the MeOH extract are regarded of special pharmaceutical interest, exhibiting, among others, antitumor, antioxidant and anti-inflammatory activities [31,32,33,34]. At this point, it is worthwhile to report that 3-formylpyrrole (**14**) is reported for the first time in genus *Pleurotus*. However, similar pyrrole alkaloids, such as *N*-[4-(2-formyl-5-hydroxymethylpyrrol-1-yl)-butyl]-acetamide and *N*-[5-(2-formyl-5-hydroxymethyl-pyrrol-1-yl)-pentyl]acetamide have been isolated from an endophytic Ascomycetes, *Fusarium incarnatum* [35,36].

In addition, 4-hydroxybenzoic acid (**15**) constitutes a common phenolic secondary metabolite of higher fungi. Interestingly, a potential biosynthetic route for the production of 4-hydroxybenzoic acid is via the bioconversion of L-phenylalanine, an aromatic amino acid present in the nitrogen source used for the biomass production in this study (corn steep liquor), and which is utilized satisfactory by *P. ostreatus*. L-Phenylalanine can be deaminated to *trans*-cinnamic acid by a phenylalanine ammonia lyase. *trans*-Cinnamic acid can be subsequently hydroxylated to β-hydroxyphenylpropionic acid, which in turn can be converted via a β-oxidation step to benzoic acid and then to 4-hydroxy-benzoic acid by the action of lignin peroxidase, an enzyme present in *P. ostreatus* cultures [37]. Even though 4-hydroxybenzoic acid has been formerly reported in many *Pleurotus* mushrooms [25], its presence in *P. ostreatus* is reported for the first time in this work. Finally, it is worth observing that nicotinic acid (**17**) and nicotinamide (**18**) have been previously identified in *Pleurotus* mushrooms, including *P. ostreatus* [38,39].

## 3. Experimental

### 3.1. General

All compounds were identified by means of spectral data (^1^H-NMR and 2D NMR), HRMS and direct comparison with the respective literature data. ^1^H-NMR (600 MHz) data were recorded on a Bruker Avance III 600 spectrometer with CDCl_3_ and CD_3_OD (Aldrich) as solvent and TMS as an internal standard. The 2D-NMR experiments (HMQC and HMBC) were performed using standard Bruker microprograms. Mass spectrometry APCI-HRMS were run on a LC/MS Thermo Scientific LTQ Orbitrap Discovery mass spectrometer. The chromatographic separation by means of preparative HPLC was applied in a Thermo Finnigan HPLC system (ThermoFinnigan, San Jose, USA), connected to a SpectralSystem UV2000 PDA detector with the incorporation of a Supelco A23, Discovery HS-C18 column (250 mm × 21.2 mm, 5 μm), while ChromQuest 2.1 software was used for the management of the data. The mobile phase composed of H_2_O–ΑCN (98:2→2:98) was gradient at a flow-rate of 1.2 mL/min and injection volume was 10 μL. The effluent was monitored at 254 nm and peak fraction was collected according to the elution profile. 

### 3.2. Biological Material

The strain used in this study was the ATHUM 4438 of *P. ostreatus* obtained from the ATHUM Culture Collection of Fungi of the National and Kapodistrian University of Athens. The stock cultures of both strains were maintained on a potato dextrose agar (PDA) slants. Slants were inoculated, incubated at 25 °C for 7 days and then stored at 4 °C.

### 3.3. Media and Fermentation Conditions in 20-L Bioreactor

**Figure 4:**
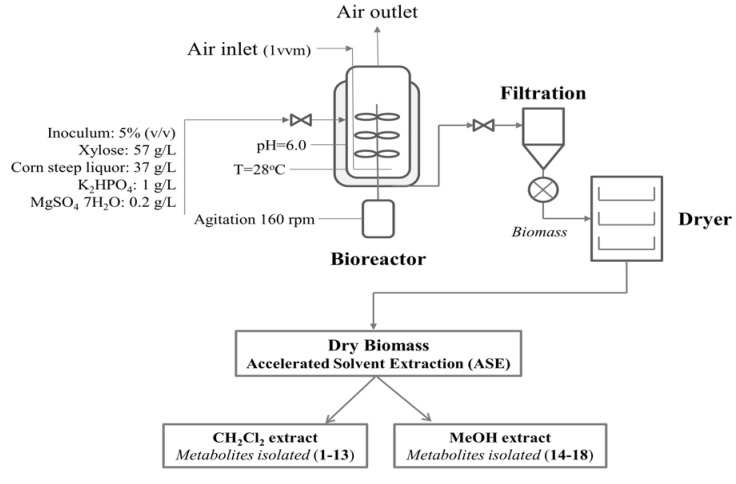
Flow diagram of conventional batch reactor system for maximum biomass production of *P. ostreatus.*

The microorganism was initially grown on PDA medium in a Petri dish, and then transferred to the culture medium by punching out 5 mm of the agar plate culture with a sterilized self-designed cutter. The composition of culture medium and fermentation conditions in the bioreactor used were the suggested for maximum biomass production, reported in our previous study [6]. The mycelial biomass was harvested, centrifuged, freeze-dried (yield of dry biomass 600 g) and powdered to ~1 mm particle size before analysis. A flow diagram of this bioprocess is shown in Figure 4.

### 3.4. Extraction

The extraction of the biomass powder (200 g) of *P. ostreatus* ATHUM 4438 was performed using an accelerated solvent extractor (ASE 300, DIONEX Co.) with cyclohexane, DCM, MeOH and H_2_O, consecutively. The sample was loaded into the stainless steel extraction cells, filled with solvents for about 1 min, and pressurized for about 5 min; After a pressure of 105 bar was obtained, heating was commenced for 20 min (static time) whilst the pressure was maintained at 90–130 bar. Extraction temperatures of 40, 40, 70, 50 °C were assigned, respectively; The unit could be flushed with fresh solvent (60 mL) pumped through the sample and entire pathway within 60 s. Purging was conducted for 1.5 min between extractions, often with nitrogen gas. The extraction for each solvent was repeated three times. The final extracts were collected in clear glass vials (250 mL). The total extraction time consumed was 80 min for each extract.

### 3.5. Isolation and Identification of Compounds

The crude DCM extract (1.5 g) was subjected to MPLC carried out with a Sepacore MPLC system (Büchi C-650 pump) on normal phase silica gel (Merck 0.02–0.04 mm) with a maximum applied pressure of 10 bar. A glass column was used (dimensions 26 mm × 460 mm), the elution was conducted with a step gradient CH_2_Cl_2_/MeOH (100:0, 95:5, 90:10, 80:20, 50:50), the solvent flow rate was set at 20 mL/min and the solvent volume for each step was 300 mL. Preparative MPLC yielded a total of 9 combined fractions: A1 (9.4 mg), A2 (231 mg), A3 (66.9 mg), A4 (47.5 mg), A5 (100.7 mg), A6 (16.8), A7 (176.9 mg), A8 (425.2 mg) and A9 (249.4 mg).

The chemical composition of non-polar fractions A1-A3 was analyzed using a previously described GC/MS method [40], resulting in the identification of compounds **1**–**8** by comparison of their retention indices (Kovats indices) with those of standard compounds and also by comparison of their mass spectra with those of a MS database (Wiley 275).

Fractions A5–A6 were combined and submitted to Sephadex LH-20 (Aldrich) column chromatography (dimensions 23 mm × 450 mm) and eluted with MeOH (100%) and solvent flow rate 3 mL/min, yielding compounds **9** (6.7 mg), **10** (4.93mg), **11** (4.36 mg) and **12** (4.5 mg). Finally, fraction A7 was chromatographed on MPLC (Buchi PP Cartridge with dimensions 12 mm × 150 mm) and eluted with a step gradient of CH_2_Cl_2_/MeOH (100:0, 95:5, 90:10, 80:20, 50:50) and afforded compound **13** (7.9 mg). The maximum applied pressure was 10 bar, the solvent flow rate was set at 20 mL/min and the solvent volumes for each step was 120 mL.

The MeOH extract (45.4 g) was subjected to adsorption chromatography using as sorbent resin XAD-4 (purchased from Rohm and Haas). The sample to be separated was diluted with distilled water. The corresponding solution was loaded on a glass column (dimensions 23 mm × 450 mm) filled with 15 g of XAD-4 (Rohm and Haas) resin previously activated with sequential passing of 30 mL MeOH and 30 mL of distilled water and repeated three times. The flow rate was set at 1.5 mL/min. The column was then washed with water to remove non-phenolic compounds. The phenolic fraction was then collected with elution of the column with 60 mL of MeOH at a flow rate of 2.0 mL/min, and the solvent was evaporated under vacuum at 40 °C until dryness. The phenolic fraction (5.8 g) obtained from this procedure was submitted to FCPC which was performed on a Kromaton FCPC unit with 1 L column capacity. The fractionation was run in dual mode using as the two-phase solvent system of EtOAc-BuOH-EtOH-H_2_O (3:10:5:15). In more details, for the preparation of the two-phase solvent system, each solvent was added to a separatory funnel and equilibrated at room temperature. The two phases were separated and the sample to be injected into the column was dissolved in 50 mL of the lower phase. The stationery phase consisted of the lower phase (polar) was pumped at a flow rate of 15 mL/min. Subsequently, the rotation was set at 950 rpm and the upper (mobile) phase was pumped at a flow rate of 8 mL/min in the descending mode. FCPC analysis yielded a total of combined fractions: B1 (77 mg), B2 (641.6 mg), B3 (304 mg), B4 (187.9 mg), B5 (313.6 mg), B6 (459.5 mg), B7 (789.2 mg), B8 (775.7 mg), B9 (397 mg) during ascending mode and fractions B10 (357.4 mg), B11 (395.9 mg), B12 (217.7 mg) and B13 (590.7 mg), during descending mode. 

Further chromatographic separation of fraction B3 by Sephadex LH-20 column chromatography (dimensions 36 mm × 450 mm) in which elution was carried out with MeOH (100%), and solvent flow rate 3 mL/min, resulted in the isolation of compounds **14** (5.2 mg) and **15** (6.8 mg). Fraction B4 was further purified by means of reversed phase preparative HPLC to afford compound **16** (13 mg) and **13** (14.5 mg). Furthermore, fraction B5 derived from FCPC analysis, was subjected to Sephadex LH-20 column chromatography (dimensions 36 mm × 450 mm), eluted with MeOH (100%) and solvent flow rate 3 mL/min, to afford compounds **18** (14.7 mg) and **19** (11.5 mg). Generally, all fractions were initially analyzed by TLC. Precoated TLC silica 60 F_254_ plates (Merck) were used (0.25 and 2 mm layer thickness for analytical and preparative TLC, respectively). Spots were visualized using UV light, and vanillin-sulphuric acid reagent. 

## 4. Conclusions

In conclusion, the findings of this study are demonstrated to be valuable, as the established fermentation process of the edible *P. ostreatus* ATHUM 4438 on bioreactor scale led to the formulation and production of already known bioactive metabolites such as fatty acids, phenolic metabolites, nucleotides and alkaloids occurring in the respective fruitbodies. Thus, the submerged fermentation of the mycelium proved to be a promising alternative for production of bioactive and nutritionally functional compounds on an industrial scale, as it is a faster, easily controlled, more efficient and of greater interest in food and drug industry process, than the established field-cultivation of the fruitbodies. 

Besides, to the best of our knowledge, the presence of *trans*-3,4-dihydro-3,4,8-trihydroxynapthalen-1(2*H*)-one, indolo-3-carboxylic acid, 3-formylpyrrole and 4-hydroxybenzoic acid in *P. ostreatus* are reported in this work for the first time, indicating that the type of mushroom cultivation probably becomes the driving force in directing secondary metabolite synthsesis. This aspect is probably explained by the fact that the mycelium was grown *in vitro* under favourably controlled conditions that hardly stimulate the conditions in nature. Additionally, the isolation and identification of metabolites that have not previously detected in fruitbodies of the studied mushroom strain may be attributed to the fact that the mycelium represents the vegetative phase, whereas the fruitbody represents the reproductive phase of the life cycle of the Basidiomycetes. Finally, it is important to mention that this report of the above metabolites confirms submerged fermentation as a suggesting option for the production of bioactive natural products by mushrooms.
